# Shock Secondary to Aortic Root Abscess With Sterile Blood Culture

**DOI:** 10.7759/cureus.15262

**Published:** 2021-05-26

**Authors:** Muhammad Z Khan, Sona Franklin, Shaheer Zahid, Steven Kutalek, James Kilcoyne

**Affiliations:** 1 Internal Medicine, St. Mary Medical Center, Langhorne, USA; 2 Medicine, Saint James School of Medicine, Park Ridge, USA; 3 Cardiology, St. Mary Medical Center, Langhorne, USA

**Keywords:** aortic root abscess, cardiogenic shock, secondary shock, sterile blood culture, transthoracic echocardiogram, aortic abscess, shock, retrograde flow, septic shock, echocardiogram

## Abstract

Shock caused by an aortic root abscess is a rare phenomenon. Due to its rarity, it is commonly not diagnosed on time to have a favorable prognosis for the patient. Our case involves an 80-year-old male presenting with leukocytosis, lactic acidosis, and hypoglycemia. Initial studies were not fruitful in determining the cause of septic shock. However, an echocardiogram conducted to clarify the finding of a non-ST segment myocardial infraction led to the incidental finding of an aortic root abscess with retrograde flow, suggesting a perforated abscess without endocarditis. Though the patient expired on day seven, our case demonstrates the importance of echocardiography in diagnosing an aortic root abscess in cases with a sterile blood culture and uneventful initial lab investigations.

## Introduction

Aortic root abscess is a complication associated with a high risk of morbidity and mortality. It is more frequently found among intravenous drug users and patients with prosthetic valve replacements [1]. Aortic root abscesses are rare causes of endocarditis; they are usually diagnosed during autopsy or surgery. *Staphylococcus *is the most common organism that causes abscesses [2]. Anguera et al. [3] found that 22% of patients with aortic infective endocarditis developed an abscess from coagulase-negative *Staphylococcus*, whereas patients without an abscess were more likely to have a streptococcal infection. They also found that patients with abscesses were more likely to undergo surgery and have a higher mortality rate.

Though there are four major categories of shock, patients with an aortic root abscess most commonly present with septic shock, which can then lead to cardiogenic shock in patients with poor cardiac output [4]. During severe septic shock, there is a high probability of a negative blood culture (up to 80% in certain studies), and in cases of a coagulase-negative pathogen, it can be even more difficult to diagnose shock from the blood culture. Therefore, it is essential to conduct further diagnostic investigations to determine the underlying pathology [5]. In this report, we present a case in which shock was due to an aortic root abscess with a sterile blood culture.

## Case presentation

**Figure 1:**
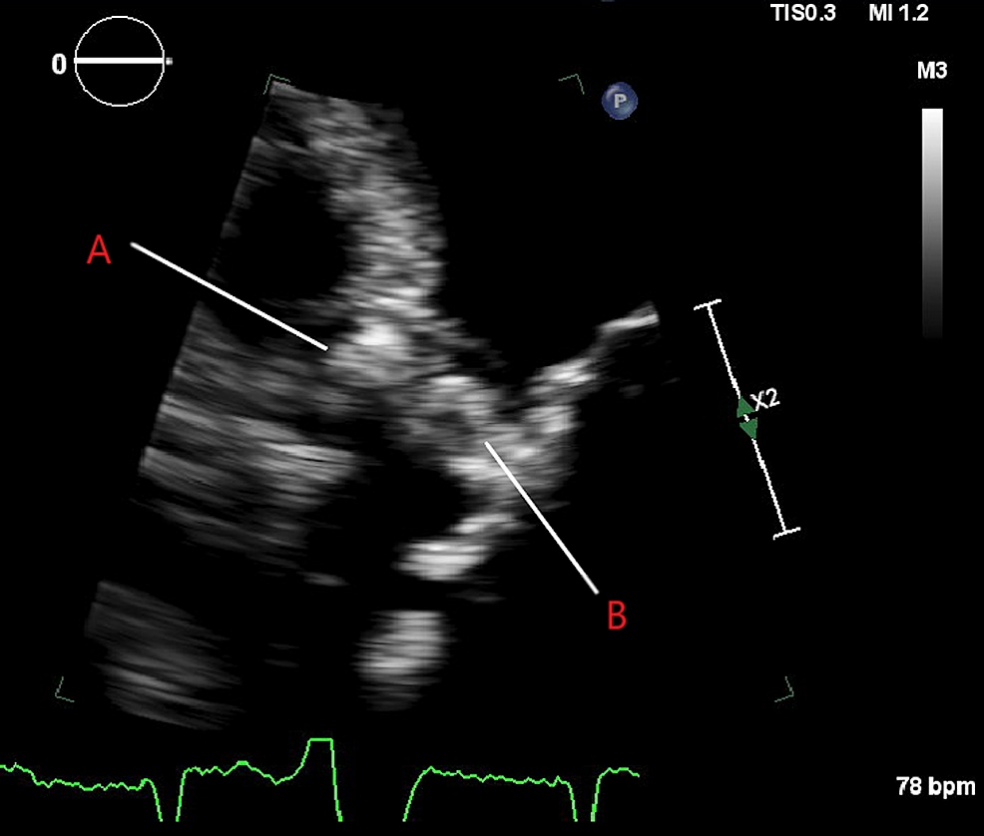
The two-dimensional aortic valve annulus abcess. A: Aortic valve annulus abscess; B: Aortic valve leaflets

**Figure 2:**
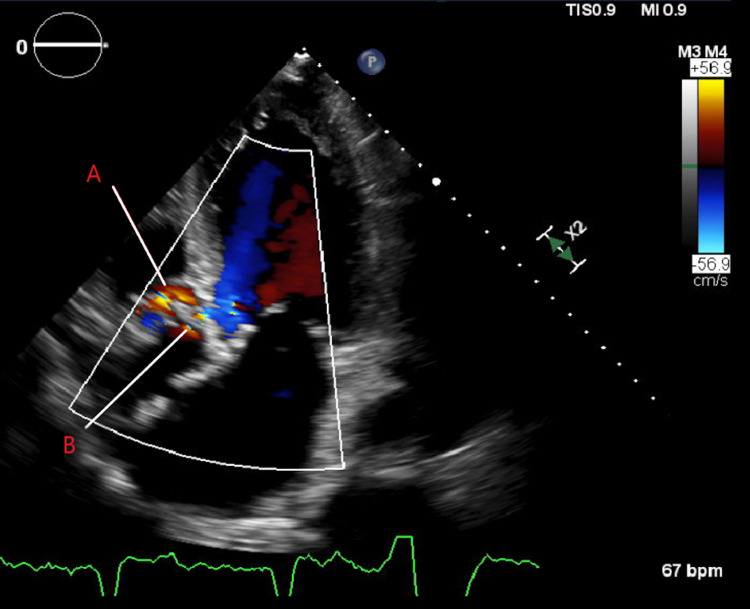
Color flow of aortic valve annulus. A: Color flow of flow through aortic annulus abscess; B: Intravalvular aortic regurgitation

An 80-year-old male had a past medical history of coronary artery disease, prior non-ST segment myocardial infarction, atrial fibrillation with a pacemaker, anomalous coronaries, severe aortic stenosis, systolic heart failure, abdominal aortic aneurysm, recent initiation of hemodialysis for end-stage renal disease, chronic obstructive pulmonary disease, and a 68 pack-year smoking history. He was initially admitted to the hospital for weakness and confusion. Hemodynamics, along with biochemical investigations, confirmed that the patient was in a state of shock. The initial differential diagnosis of shock was cardiogenic versus septic shock because there was severe aortic stenosis and reduced left ventricular ejection fraction. Fever, leukocytosis, lactic acidosis, and hypoglycemia were noted in the emergency department. Transaminase elevation was also seen, so there was concern about progressive liver failure. A computed tomography (CT) scan of the brain and chest X-ray were unremarkable. He had an altered mental status and metabolic acidosis. Later, he developed respiratory distress and required intubation. A transthoracic echocardiogram showed a left ventricular ejection fraction of 25% and suspicion of an aortic root abscess with retrograde flow suggesting a perforated abscess (Figures 1, 2). The left ventricular ejection fraction was similar to one from a year earlier, but the findings concerning the abscess were new. No valvular vegetation was present that would be consistent with traditional infective endocarditis. A transesophageal echocardiogram was recommended to definitively diagnose the aortic root abscess; however, due to his poor prognosis, his family requested comfort measures only. The patient expired on hospital day seven.

## Discussion

The patient in this case report was an 80-year-old male with multiple comorbidities who was found to have an aortic root abscess. Initial investigations with blood laboratory tests, including blood cultures, chest X-ray, and urine analysis were not fruitful in determining the etiology of sepsis. Vasopressors and piperacillin-tazobactam were initially administered to the patient to treat underlying cardiogenic or septic shock, although the source was not clear. An echocardiogram was performed within 24 hours of admission, and it showed an aortic root abscess, which was determined to be the source of sepsis. In rare cases in which the source of infection is unknown, a transthoracic echocardiogram is needed as an initial diagnostic test to diagnose and identify the location of the infection in the heart or aorta.

In a study conducted by Mahmoud et al. [1], it was observed that patients with underlying prosthetic valve replacements and septic shock had significantly higher rates of aortic root abscesses. Among patients with aortic root abscesses, there was an increased incidence of sterile blood cultures and a low likelihood of cardiac vegetation; both of these characteristics were present in this patient. In their study, Mahmoud et al. [1] reported that echocardiographic data was the most significant determining factor in identifying the shock from the abscess.

A limitation of this case report is that a transesophageal echocardiogram could not be conducted due to the decision of the family. While the goals of the care discussion with the family led to the patient receiving comfort measures only, any patient in septic shock with an aortic root abscess requires surgical management [6]. Surgical management itself carries a high mortality rate; Kirali et al. [6] found a 22.2% in-hospital mortality rate during their 15-year experience managing aortic root abscesses. The timing of diagnosis played a major role in improving the surgical prognosis for patients. Therefore, we believe it is of utmost importance that transesophageal echocardiogram is performed as an initial diagnostic measure for shock patients with sterile blood cultures to diagnose a rare condition, such as an aortic root abscess in this case.

## Conclusions

This case demonstrates the importance of performing a transesophageal echocardiogram to rule out the rarer causes of shock secondary to aortic root abscess, particularly in the absence of a positive blood culture. Given the poor prognosis associated with an aortic root abscess, this case report aimed to shed light on an uncommon cause of shock.
